# Retention of Fœtus Several Months after Its Death*Several of these singular cases are on record.—Ed. Jour.

**Published:** 1861-04

**Authors:** J. Tripp

**Affiliations:** Morenci, Mich.


					﻿RETENTION OF FCETUS SEVERAL MONTHS AFTER
ITS DEATH. *
* Several of these singular cases are on record.—Ed. Jour.
UY J. TRIPF, 31. U., Morenci, Mich.
I was called Feb. 2d, 1861, at 9 o’clock P. M., to attend
Mrs. D. in her third confinement. Three hours after she was
delivered of a full grown female child, healthy in every re-
spect. Ten minutes after she was delivered of a second which
was dead. From the appearance I should think that the
death took place about the fifth or sixth month of gestation.
The mother and living child did well.
The singularitity of the case is that the death and long
retention of the foetus were accompanied by no unusual
symptoms.
				

